# Optimizing online teaching effectiveness in elementary education: Exploring multifaceted pathways based fsQCA analysis

**DOI:** 10.1371/journal.pone.0345463

**Published:** 2026-03-23

**Authors:** Xiaofang Ma

**Affiliations:** Department of Education, Fuzhou University of International Studies and Trade, Fuzhou, China; RMIT University, VIET NAM

## Abstract

The effectiveness of online teaching has emerged as a critical concern during the COVID-19 pandemic, with a particular emphasis on fostering a sustained interest in online instruction among educators. This study employs fuzzy-set qualitative comparative analysis (fsQCA) to investigate the complex causal relationships that underpin the online teaching efficacy of elementary school teachers. A comprehensive questionnaire was administered to 171 elementary school teachers, capturing their perspectives across six key dimensions: the acceptability of online teaching, teachers’ Technological Pedagogical Content Knowledge (TPACK), students’ autonomous learning, teacher-student interaction, course design and implementation, and technological support for teaching. The findings reveal a multiplicity of configurations that contribute to enhanced online teaching effectiveness, with teachers’ acceptance of online teaching and their TPACK being identified as central conditions, pivotal for the improvement of online teaching effectiveness.

## 1. Introduction

The efficacy of instruction is a crucial determinant of students’ academic performance. The disruption caused by the COVID-19 pandemic has led to the widespread adoption of hybrid teaching approaches, blending in-person and traditional classroom learning with online instruction. Consequently, the effectiveness of online teaching has rapidly emerged as a focal point in the field of elementary education—an area where young learners’ limited self-regulation and dependence on guided learning make high-quality virtual instruction particularly critical [1] Prior studies have offered mixed insights: Totlis [2] conducted a comparative analysis of traditional teaching methods and three distinct distance learning models, finding the latter to be more efficacious in specific contexts, while Afzal et al. [3] discovered that a notable number of educators remained dissatisfied with their e-learning platforms. These findings collectively suggest that while virtual instruction may not universally match the effectiveness of in-person teaching, it has become an indispensable contingency plan during emergencies and a potential long-term supplement to traditional education [1,4]. Thus, enhancing the effectiveness of online teaching in elementary education is not only imperative for addressing immediate pandemic-related challenges but also for shaping the future of inclusive, flexible education—requiring sustained engagement with virtual instruction beyond crisis periods [5].

### 1.1. Research questions

Despite growing attention to online teaching effectiveness (OTE) in elementary education, three interconnected problems persist in existing research, hindering the development of targeted improvement strategies. First, the causal complexity of OTE remains underexplored: OTE is shaped by intertwined human factors (e.g., teacher attitudes, student autonomy, teacher-student interaction), curricular factors (e.g., course design), and technological factors (e.g., technical support), yet most studies treat these variables as isolated predictors rather than synergistic components of a system [6,7]. This oversimplification fails to capture how combinations of factors—rather than individual variables—drive successful online instruction, especially for elementary students with unique developmental needs. Second, methodological limitations restrict insight into multi-factor interactions: Prior research has predominantly relied on regression modeling and correlation analyses, which excel at identifying linear relationships between single variables and OTE but struggle to unpack non-linear, configurational pathways (e.g., whether strong teacher-student interaction can compensate for limited technical support) [8]. Third, elementary education-specific gaps remain unaddressed: Most OTE research focuses on higher education or secondary education, where learners exhibit greater self-direction; few studies tailor their analysis to elementary settings, where teachers’ ability to adapt technology to young learners’ needs (e.g., simplified tools, structured guidance) and parental/teacher support play outsized roles [9]. Together, these gaps leave policymakers and elementary educators without clear answers to a critical question: What combinations of factors (teacher, student, curricular, technological) consistently lead to high OTE in elementary education, and which factors are most essential to prioritize?

### 1.2. Research gaps

Building on the above problematization, three key research gaps are identified to frame the scope of this study:

Gap 1: Neglect of configurational causality in elementary OTE: Existing studies on elementary online teaching focus on independent variable effects (e.g., “does teacher TPACK correlate with OTE?”) rather than exploring how factor combinations (e.g., “TPACK + course design + technical support”) produce OTE. This ignores the reality that no single factor is sufficient to guarantee effectiveness, and contextual trade-offs (e.g., strong course design mitigating weak student autonomy) are common in practice [10].

Gap 2: Limited application of complexity-sensitive methods in elementary education: While fuzzy-set Qualitative Comparative Analysis (fsQCA)—a method designed to identify causal configurations—has been used to study OTE in higher and vocational education [11], it remains underutilized in elementary education. This limits our ability to capture the context-specific, multi-pathway nature of OTE for young learners.

Gap 3: Unresolved inconsistencies in factor importance: Literature debates the necessity of key factors (e.g., some studies frame student autonomous learning as critical for OTE [12], while others emphasize technical support [13]. Without a configurational lens, these inconsistencies cannot be resolved—leaving educators unsure which factors to prioritize when resources are limited.

### 1.3. Research contributions

This study addresses the above gaps and makes three distinct contributions to the field of elementary online education research:

Methodological contribution: It applies fsQCA to elementary OTE research, moving beyond linear, variable-centered approaches to unpack the non-linear, configurational pathways that drive effectiveness. This method enables the identification of “core conditions” (universally essential factors) and “peripheral conditions” (context-dependent supplements)—a distinction that traditional methods cannot capture [14].

Empirical contribution: It provides evidence from 171 elementary school teachers across diverse grades and subjects, identifying specific, actionable factor combinations (configurations) that lead to high OTE. This resolves prior inconsistencies by showing when factors like student autonomy or technical support are necessary (or unnecessary) for effectiveness.

Practical contribution: It offers context-specific guidance for elementary schools with varying resources (e.g., urban vs. rural, well-funded vs. resource-constrained). By distinguishing core from peripheral conditions, the study helps educators prioritize investments (e.g., teacher TPACK training over expensive technical tools) rather than adopting “one-size-fits-all” solutions.

To address the identified research problem and gaps, this study has two primary research objectives:

To explore the multifaceted configurational pathways to high OTE among elementary school teachers, with a specific focus on six key influencing factors identified through literature synthesis: teachers’ acceptability of online teaching (AFT), teachers’ Technological Pedagogical Content Knowledge (TPACK), students’ autonomous learning (SAL), teacher-student interaction (TSI), course design and implementation (CDI), and teaching technological support (TTS).

To identify the core and peripheral conditions within these configurational pathways using fsQCA, clarifying which factors are universally essential for enhancing OTE and which function as context-dependent supplementary conditions—ultimately providing targeted, actionable strategies for elementary schools to optimize the quality of online teaching in both emergency and routine educational scenarios.

## 2. Literature review

### 2.1. Introduction to factor analysis in online education

Prior studies have explored diverse aspects of the factors impacting the efficacy of online education. For instance, Jui-Che Tu [13] identified variables such as the approval of technology models, psychological learning processes, situational learning simulation, and learning theories. Noesgaard & Rngreen [15] classified the elements influencing the effectiveness of online learning into the nature of online learning solutions and processes, personal factors, and situational factors, with a specific emphasis on resources (time, technology) and support (from administrators, information technology staff or peers). Inusah and Debrah [6] primarily focused on student factors affecting teachers’ online teaching during the COVID-19 pandemic, including student interaction, access to quality networks, and students’ ability to learn online. Husin et al. [7] developed a conceptual framework that encompasses technological, learner, administrator, and personal factors that affect teachers’ effectiveness in online teaching. Martin [16] highlighted the importance of instruction, content, motivation, relationships, and mental health in online education. Prakasha [8] further categorized the factors into course design, course delivery, instructor behavior, student characteristics, and administrative support as the fundamental elements of effective online teaching.

By synthesizing the aforementioned literature, the scholar has distilled the influential factors on educators’ remote instruction into three main categories: interpersonal dynamics (encompassing teacher and student attributes, as well as teacher-student interplay), instructional content variables, and technological considerations. Meanwhile, administrative influences primarily manifest through the facilitation of technical assistance and educational resource provision for teachers engaging in online pedagogy. This investigation subsumes technical assistance under technological factors and educational resource provision under course-related considerations.

Notably, most existing studies adopt variable-centered approaches to identify independent factor effects, while configurational methods like fuzzy-set Qualitative Comparative Analysis (fsQCA) have emerged as powerful tools for unpacking synergistic factor interactions. Recent advancements in fsQCA applications across educational contexts provide critical benchmarks for positioning the current research.

### 2.2. Configurational research on online education based fsQCA

Fuzzy-set Qualitative Comparative Analysis (fsQCA) has become a pivotal methodology for exploring the complex multi-factor interactions that shape online education outcomes. Different from traditional variable-centered research that isolates the independent effects of single factors, fsQCA focuses on identifying causal configurations—the synergistic combinations of conditions that drive outcomes such as online learning engagement and teaching effectiveness. For elementary education, where young learners have limited self-regulation and rely heavily on guided learning, fsQCA is particularly suitable for unpacking the contextualized factor combinations that underpin high online teaching effectiveness (OTE). Existing fsQCA research on online education in compulsory education (K-9) provides direct theoretical and empirical benchmarks for this study, and the core insights of these studies are synthesized below to frame the research context and highlight the uniqueness of elementary education OTE research.

FsQCA applications in compulsory education (K-9) online education research consistently emphasize the centrality of age-adaptive supportive configurations, which align with the developmental characteristics of young learners and form the core theoretical basis for this study. Their findings revealed a universal core pathway for K-9 online learning engagement: the combination of strong teaching presence, perceived usefulness of online tools, and parental support, which underscores that adult guidance and structured pedagogy are non-negotiable for young learners with limited independent learning ability. For elementary students specifically, the research further found that “perceived ease of use of online tools” emerged as a core condition in most effective configurations—this is a key distinction from secondary school students, reflecting elementary learners’ limited technical autonomy and the need for simplified, age-appropriate digital teaching tools.

This conclusion is further corroborated by Zhou et al. [9], who used fsQCA to explore the influencing factors of teachers’ digital competence in Chinese compulsory education. Their study found that elementary school teachers require targeted digital training and simplified teaching tools to translate technical competence into effective online instruction; generic technology training that ignores the developmental constraints of elementary students fails to improve actual online teaching quality. Notably, Zhou et al. [9] also identified regional resource disparities as a critical contextual factor shaping the effectiveness of online education configurations in elementary settings: in eastern coastal regions with robust internet infrastructure, the combination of “high perceived utility of technology + low perceived difficulty of use” strongly predicted teachers’ digital teaching effectiveness; in western regions with limited connectivity, the same outcome required “high perceived utility of technology + institutional technical support”, meaning that infrastructure gaps must be compensated by organizational resources. This finding highlights that the configurational pathways of elementary online education are not static but are shaped by regional resource contexts, providing an important contextual perspective for this study’s exploration of elementary OTE factor combinations.

In addition to the core insights from compulsory education-focused fsQCA research, comparative references from fsQCA studies in other educational stages (higher and vocational education) further clarify the unique configurational characteristics of elementary education OTE. University-focused fsQCA research [17] identifies learner internet self-efficacy and autonomous learning ability as core conditions for high online learning outcomes, reflecting the strong self-directed learning characteristics of university students. Vocational education fsQCA research [11] prioritizes “industry-aligned interactive pedagogies” as the core of effective online teaching configurations, centered on the cultivation of practical professional skills. In sharp contrast, elementary education fsQCA research consistently finds that learner autonomy is a peripheral rather than core condition, and parental support, teacher-led structured teaching, and age-adaptive technical tools replace learner self-efficacy as the key supportive factors. This fundamental distinction confirms the necessity of conducting targeted fsQCA research on elementary education OTE—rather than extrapolating conclusions from other educational stages—and further justifies the focus of this study on elementary school teachers and the contextualized factor combinations of OTE in this specific stage.

Overall, existing fsQCA research on online education has confirmed the value of configurational analysis for exploring complex educational outcomes, and compulsory education-focused studies have revealed the core characteristics of elementary online education configurations: age-adaptive supportive conditions, the centrality of teacher and parental guidance, and the modulation of regional resource disparities. However, current research mostly focuses on student online learning engagement rather than teacher-level online teaching effectiveness, and there is a lack of targeted fsQCA exploration of the factor combinations that drive elementary school teachers’ OTE. This study fills this gap by taking elementary school teachers as the research subject and using fsQCA to identify the core and peripheral conditions of high OTE configurations, which is a natural extension and in-depth exploration of existing configurational research on elementary online education.

### 2.3. Factors influencing online teaching effectiveness

Alomari et al. [18] conducted an investigation into the determinants of learning management system use, determining that certain developing nations struggle with the adoption of this technology due to the oversight of human elements. These factors were identified through a comprehensive review of literature as technological attributes, psychological traits, and student-teacher interaction characteristics. Consequently, this research contends that human factors play a pivotal role in online education, with the key individuals being the educator and the learners. As such, this study classifies human factors as instructor-related elements, student-related elements, and student-teacher interaction.

#### 2.3.1. Teacher factors.

- Acceptability for teachers

Educators’ embrace of virtual instruction directly correlates with the efficacy of online teaching. According to Shklarski, the abrupt shift to remote instruction prompted by the COVID-19 pandemic has placed significant stress on educators, leading to a heightened need for technological and emotional support, as well as adjustments to their workload from their institutions [5]. In the initial stages of virtual teaching, many teachers exhibited apprehension, largely stemming from a lack of familiarity and proficiency [4] in online pedagogy, as well as the perception that virtual instruction would require more time for course preparation and resource procurement [4,19].

In contrast, university educators exhibited greater willingness to engage in remote instruction [4]. This is largely attributable to disparities in the student population. The limited readiness of primary school students to adapt to remote learning has caused concerns among elementary school educators regarding the efficacy of delivering instruction and learning in an online format [1]. Nonetheless, the majority of educators are willing to embrace online instruction during exceptional circumstances, despite the prevailing belief among most teachers and students that remote teaching is not as effective as in-person instruction, and is only a last resort in emergencies [1,4].

- Teachers’ TPACK

The Technology Pedagogical Content Knowledge (TPACK) framework is essential for all educators to effectively integrate technology into teaching and learning [20]. There are three fundamental components of TPACK that must be fully mastered in order to carry out any teaching and learning activities through various technological means and platforms, particularly during the COVID-19 pandemic [21] Brinkley-Etzkorn’s [22] research demonstrated that following TPACK training, teachers showed statistically significant changes in integrating elements into their course syllabi for redesign, and there was a notable enhancement in their self-reported teaching effectiveness on a subsequent survey. Afzal [3] examined the impact of an e-learning system on medical college teachers during the COVID-19 pandemic and further investigated the satisfaction of medical college teachers with the e-learning system. The findings indicated that the majority of medical college teachers (76%) were dissatisfied with the e-learning system, yet they also recognized it as the best available alternative strategy and believed that improving their computer technology skills could enhance their satisfaction with the system. Rachael et al. [23] assessed the efficacy of the components of the TPACK framework for online teaching and concluded that the synergistic effects of teachers’ content, pedagogical, and technological knowledge are indispensable for online teaching effectiveness. Collectively, these studies demonstrate that teachers’ proficiency in TPACK is a pivotal factor in the effectiveness of online teaching and learning.

#### 2.3.2. Students factor—students’ autonomous learning.

Cao et al. [12] conducted interviews with 152 mathematics educators from 20 provinces (cities) in China and utilized the four-factor pedagogical tetrahedron framework (involving the teacher, technology, students, and mathematics) to pinpoint the challenges they faced regarding technology, teacher-student interactions, and mathematics instruction. Findings revealed that instructors perceived the effectiveness of online teaching hinged largely on students’ self-regulation. This places a significant emphasis on students’ independence, which may be inadequate to support autonomous online learning for younger elementary school students. However, the majority of educators are committed to enhancing the efficacy of online instruction through various means. Students have the capacity and necessity to learn without direct teacher supervision. Educators should encourage students to self-regulate, while also working to establish a distinctive online presence, different from their physical presence on campus [24]. Furthermore, educators must acknowledge that learners’ ability for independent learning is continuously evolving and that online courses can bolster students’ problem-solving, critical thinking, and adaptability as their autonomy expands [25].

#### 2.3.3. Teacher-student interaction.

Capperucci’s research demonstrated a strong association between educators’ implementation of interactive methodologies and academic achievements among students [26]. Khan investigated the effects of five key factors, encompassing adaptability, cost-effectiveness, connectivity, comprehension, and engagement, on virtual instruction amidst the pandemic, revealing that all factors have a beneficial impact on the efficiency of online learning [27].

In the virtual learning environment, teachers take on the role of a facilitator and mentor, while students are expected to be more proactive and engaged. Rather than merely following instructions, students are responsible for driving their own learning [28]. Utilizing a range of pedagogical approaches such as lectures, case studies, debates, discussions, experiential learning, brainstorming sessions, and games, as well as incorporating drills and other instructional methods, is essential in the online classroom [25]. The use of visual content can further enhance student engagement and interaction [29]. It is crucial to prioritize synchronized communication for immediate feedback in online learning, as this has been shown to be highly effective [24,25]. Well-implemented programs have the potential to enhance students’ problem-solving abilities, critical thinking, and adaptability.

#### 2.3.4. Course factors: Course design and implement.

The elements of a course play a crucial role in shaping the efficacy of online teaching and learning. These elements primarily encompass the structure of the online course, the pedagogical approaches employed, and the assessment methods. The design of online courses should be meticulously organized, incorporating clear channels for communication, monitoring, and feedback. Activities should be closely integrated with assessment and feedback in order to captivate students’ attention [24]. Diverse formats can be utilized to leverage the multimodal nature of online instruction, such as the integration of audio, video, text, and even gamification elements, which can greatly enrich the online learning experience [25,30]. Constructive approaches can be employed to ensure more valid assessment practices, while also empowering students to take responsibility for their own learning. Furthermore, these methods can be utilized to present real-life problems through the assignment of authentic tasks [30]. Assessments necessitate the use of materials and techniques that differ from those used in traditional settings in order to be truly effective [31].

Shklarski proposed that there is a necessity for comprehensive institutional support to enhance online instructional techniques, not only to address the societal repercussions of COVID-19, but also to enable educators to appreciate the advantages of online teaching and to be motivated to continue teaching in the virtual realm in the future [5]. Amidst the COVID-19 pandemic, Capperucci conducted a survey of over 16,000 teachers in Italy to reexamine instructional approaches and tactics [26]. The research revealed a positive association between educators’ utilization of interactive teaching strategies and alternative evaluation activities, and heightened perceptions of student learning outcomes and satisfactory performance assessment. It is contended that training in online teaching is valuable only when it is focused on pedagogy and assessment approaches in digital learning environments, rather than merely on technology [26].

#### 2.3.5. Technological factor—teaching technological support.

Numerous researchers have incorporated technological elements into their investigations of the factors influencing online instruction and learning. They maintain that technological components are crucial for facilitating the smooth operation of online teaching and learning [6,7,13,15]. Shklarski discovered that the sudden shift to remote teaching prompted by COVID-19, a stress-inducing situation, has left teachers in dire need of both technological and emotional support [5]. Nevertheless, technical assistance alone cannot guarantee the efficacy of online teaching, as Capperucci contended that training for online instruction is only beneficial when it focuses on teaching and assessment methods in virtual learning environments, rather than solely on technology [26]. Thus, technology is a necessary but insufficient prerequisite for online teaching. Furthermore, the role of technology varies across different subject areas. Cao et al. examined the challenges facing elementary school mathematics in online instruction and asserted that the interactive capabilities of technology and platforms should be enhanced to better accommodate the diverse needs of various subject areas in online teaching [12].

## 3. Research model

This study utilized the Community of Inquiry (CoI) framework as a theoretical basis to construct a fsQCA research model, drawing from scholarly literature and the intelligent education framework [32]. Additionally, it integrated insights from Ma’s qualitative research on the factors influencing the efficacy of educators’ online instruction [1]. The subjects involved in online education were categorized into human factors (involving educators, learners, and educator-learner interactions), course-related factors, and technological factors. Educator factors encompassed acceptance of online instruction and educators’ technological pedagogical content knowledge (TPACK); learner factors encompassed student self-directed learning; and educator-learner interaction referred to the interaction between educators and learners in an online learning environment. Course-related factors were condensed into course design and implementation, encompassing the structuring and organization of online courses, as well as the instructional and assessment methodologies employed. Technological factors primarily pertained to technical support.

**Fig 1:**
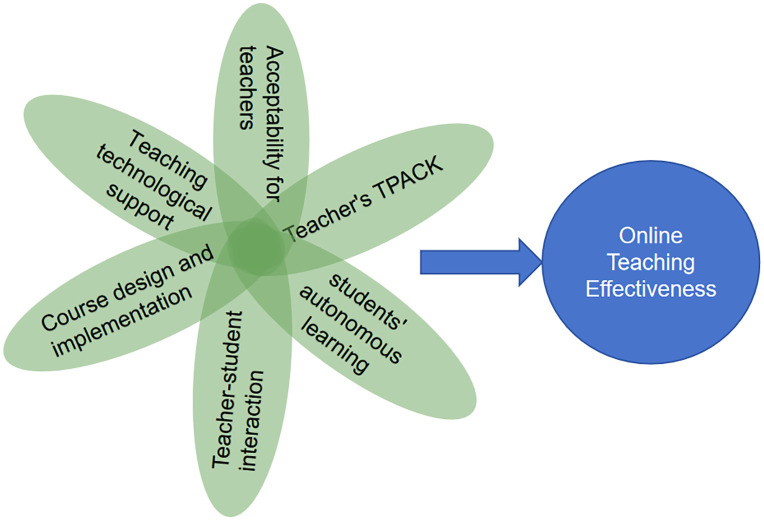
Conceptual model of online teaching effectiveness. This conceptual model illustrates the key antecedent factors and their direct associations with online teaching effectiveness, including teachers’ acceptability for teachers, teaching technological support, online course design and implementation, students’ autonomous learning and teachers’ technological pedagogical and content knowledge (TPACK). All identified factors are posited as core drivers of the effectiveness of online teaching practices.


As a result, the present study established a theoretical framework encompassing educators’ approval (AFT), educators’ technological pedagogical content knowledge (TPACK), learners’ self-directed learning (SAL), educator-learner engagement (TSI), curriculum planning and execution (CDI), and educational technology assistance for educators (TTS) as the predictor variables and the efficacy of virtual instruction as the outcome variable (see Fig 1), as follows:

OTE=f (AFT, TPACK, SAL, TSI, CDI, TTS,)

## 4. Methodology

### 4.1. Data collection and respondents

This study employed snowball sampling as the core recruitment method, supplemented by questionnaire distribution via official primary school education platforms and teacher professional learning communities. This sampling strategy was strategically adopted for three key reasons with solid methodological and practical rationales: First, elementary school educators form a relatively bounded professional community with strong internal connectivity (e.g., through regional teaching and research groups, inter-school cooperation projects, and online teacher communication clusters), and this inherent social network of the group enables snowball sampling to effectively reach participants from diverse geographical contexts (including urban, suburban and rural areas) and various school types (such as public primary schools, private primary schools and rural central primary schools). This advantage of snowball sampling in accessing geographically and institutionally diverse samples within a bounded professional group has been verified and supported by existing educational research [33,34]. Second, given the study’s focus on “online teaching effectiveness in elementary education”—a research topic requiring participants with direct and practical online teaching experience—snowball sampling allowed initial participants (frontline elementary school teachers with rich online teaching practice) to refer peers with relevant professional experience, which effectively ensured the phenomenological relevance and representativeness of the research sample. Third, this method effectively addressed the practical challenge of accessing the scattered elementary school teacher population in China’s educational context, particularly in rural or suburban areas where direct recruitment via public channels (e.g., social media and public recruitment platforms) is inefficient due to low participation willingness and information asymmetry.

The Questionstar platform (https://www.wjx.cn) was chosen for questionnaire distribution due to its widespread adoption in Chinese educational research and robust functions for response quality control (e.g., setting response time thresholds, logic verification for contradictory answers).

The questionnaires were distributed on August 7, 2023, and by the deadline of September 2, a total of 182 questionnaires were collected. All participants provided written informed consent. To ensure the reliability of the surveys, 171 surveys remained after excluding those with a response time of less than 80 seconds, all of which were found to be valid. This sample size is sufficient for the fuzzy-set qualitative comparative analysis (fsQCA), which consistent with Fiss [10], fuzzy-set qualitative comparative analysis (fsQCA) does not require large sample sizes like regression analysis, as it focuses on unpacking causal configurations rather than linear correlations, thereby supporting small-to-medium sample research. Regarding the gender distribution of the respondents, 11.54% were male educators and 88.46% were female educators, which aligns with the general gender distribution of elementary school teachers in the nation. With respect to the age distribution of the teachers, 28.70% were between the ages of 22–30, 36.2% were between 31–40, 22.7% were between 40–50, and 12.4% were 50 or older. In terms of grade level, 26.37% of the teachers taught grades 1–2, 33.52% taught grades 3–4, and 40.11% taught grades 5–6. When it comes to the subjects taught, the majority of teachers (39.01%) were Chinese teachers, followed by mathematics teachers (26.37%), and the remaining subjects included science, art, music, information technology, physical education and health, morality and rule of law, English, and psychology (Table 1). The mean, standard deviation, maximum, and minimum values for each variable are displayed in Table 2.

**Table 1:** Demographic characteristics of the sample.

Demographic Characteristics	Demographic Dimension	Proportion
Gender	male	11.54%
	female	88.46%
Age	22-30	28.7%
	31-40	36.2%
	41-50	22.7%
	>50	12.4%
Grade taught	Grade 1–2	26.37%
	Grade 3–4	33.52%
	Grade 5–6	40.11%
Subject taught	Chinese	39.01%
	mathematics	26.37%
	other	34.62%

**Table 2:** Descriptive statistical analysis of the variables.

Variable	Mean	Standard deviation	Minimum	Maximum	Number of cases	Missing
Acceptability for teachers	3.3187	0.942	1.000	5.000	171	0
Teachers’ TPACK	3.5791	0.622	1.000	5.000	171	0
Students’ autonomous learning	3.0263	0.771	1.000	5.000	171	0
Teacher-student interaction	2.3888	0.845	1.000	5.000	171	0
Course design and implementation	3.0311	0.729	1.000	5.000	171	0
Teaching technological support	2.5292	0.874	1.000	5.000	171	0

### 4.2. Ethical statement

This study was approved by the Institutional Ethics Committee of Fuzhou University of International Studies and Trade (FW-2023-0021, July 24, 2023) and written informed consent was obtained.

### 4.3. Measurements checking

The survey utilized in this investigation comprises three sections: the initial part encompasses demographic details of the participants, the second part consists of a questionnaire encompassing six factors within the research model, and the final part incorporates an online teaching efficacy survey. The first segment of the survey gathers data regarding the respondents’ gender, age, teaching grade level, and subject area. The second section of the survey incorporates the TPACK questionnaire developed by Schmidt et al. [35] along with additional questionnaires for various factors developed by the researcher. The third part, the online teaching efficacy questionnaire, was adapted from the teaching efficacy questionnaire by Garrison [36]. The entire survey consists of 53 items. To ensure the reliability and validity of the survey, a Cronbach’s alpha coefficient was utilized for the reliability test, and KMO and Bartlett’s test were used for the validity test.

During the assessment of reliability, the Cronbach’s alpha coefficient was found to be 0.964, surpassing the threshold of 0.8, thus indicating favorable reliability of the questionnaire. Subsequently, the questionnaire was subjected to a validity test, with KMO and Bartlett’s test yielding a KMO value of 0.917, surpassing the 0.8 benchmark and indicating suitability for information extraction. In Bartlett’s test for sphericity, the P-value was 0.000, below the 0.05 threshold (Table 3), further confirming the questionnaire’s good validity.

**Table 3:** Results of the reliability and validity tests.

Tests	Number of questions	Number of cases	Cronbach’s alpha coefficient
Reliability	53	171	0.964
Validity	KMO values	0.917	
	Bartlett Sphericity Check	Approximate cardinality	40570.375
		df	1711
		p-value	0.000

### 4.4. Fuzzy-set qualitative comparison analysis (fsQCA)

Fuzzy set qualitative comparative analysis (fsQCA) is a case-oriented research method rooted in set theory and configurational thinking, which achieves an organic integration of qualitative and quantitative analysis. Its core logic is to explore the causal associations among antecedent conditions, condition combinations and outcome variables from a set-theoretic perspective by virtue of configurational theory and Boolean algebraic operations, thereby unraveling the complex causal relationships underlying educational phenomena [10,14].

Compared with traditional single case studies, fsQCA boasts prominent methodological advantages [14]: first, it adopts a multi-case research design, and fully captures the complexity and heterogeneity of individual cases by constructing a pluralistic analytical framework of causal relationships; second, it is capable of analyzing multiple causal combinations, identifying causal condition subgroups that drive the emergence of specific outcomes, exploring the equifinality among different causal condition subgroups, and separately examining the causal condition configurations corresponding to the occurrence and non-occurrence of specific outcomes; third, different from traditional qualitative research that only explores inter-case variable relationships, fsQCA takes logical condition combinations as the analytical basis, enabling comparative analysis of different scenarios within the same model and across different models.

The methodological superiority of fsQCA is more distinct when compared with traditional regression analysis that centers on independent variables and their marginal effects [14]. First and foremost, fsQCA has no stringent requirements for sample size and can systematically and effectively process multi-case comparative research data, which is particularly advantageous for small and medium-sized sample research where variables are mainly dichotomous, nominal and ordinal. This characteristic makes fsQCA break through the sample size limitation of regression analysis and become an ideal tool for exploring complex causal configurations in educational research with limited sampling conditions. Second, fsQCA focuses on analyzing the joint effect of causal condition combinations on outcomes, which makes up for the inherent deficiency of regression analysis that assumes the independence of independent variables and is susceptible to autocorrelation and multicollinearity. Third, fsQCA adheres to the perspective of causal equifinality, emphasizing that multiple distinct factor combinations may lead to the same outcome. This not only clarifies the multiple paths and mechanisms to achieve specific outcomes, but also can measure the net effect of different causal combinations on the outcome, thus enabling an in-depth and comprehensive exploration of the causal antecedents of the outcome in small and medium-sized sample analysis.

#### 4.4.1. Data calibration.

In the current investigation, we employed the data-driven method to standardize the data of these factors. The top 25% value was assigned a score of 0.95 as complete inclusion, the bottom 25% value was assigned a score of 0.05 as complete exclusion, and the middle value was assigned a score of 0.5 as an overlap.

#### 4.4.2. Truth table establishment and refinement.

*Creation of truth tables:* Following the calibration of the preceding and consequent variables, a truth table was devised, enumerating every potential logical amalgamation of the circumstances articulated in binary forms (i.e., existence or nonexistence) in accordance with the membership evaluations of the altered fuzzy set of all variables [14].

*Revise truth table:* Adjust the truth table by specifying the frequency cutoff and consistency threshold. When the total number of cases included in a study is relatively limited, the frequency threshold should be 1 or 2, but if the total number of cases is substantial, a higher threshold should be chosen [14]. Generally, the cutoff value should not be lower than 0.75; a cutoff value of ≥0.85 is recommended [14]. Consequently, in this investigation, the frequency cutoff was established at 2 and the consistency threshold was set at 0.85.

*Enhancement of the truth table:* Subsequently, the Quine-McCluskey algorithm utilizing counterfactual examination was utilized to enhance the truth table [10,14]. The impacts of circumstances on the results were defined as “existent or non-existent.” The algorithm generated three types of resolutions (elaborate resolution, economical resolution, and moderate resolution) [10,14].

*Interpretation of the resolution:* The differentiation of the central and non-central elements could be discerned by contrasting the intricate and simple resolutions within the prior framework [10,14].

### 4.5. Complementary analysis

#### 4.5.1. Sensitivity analysis.

Subsequently, a sensitivity analysis is conducted to verify the reliability of the findings through alternative condition specifications. In other words, the variables are replaced and adjusted with other rational anchor systems [10]. The study evaluates the resilience of the results by substituting the anchors with upper, middle, and lower quartiles.

#### 4.5.2. Predict validity analysis.

The predictive validity assessment was utilized to validate the capacity of the theoretical configuration model to forecast the outcome variables across diverse datasets [37].

#### 4.5.3. Post hoc analysis.

Incorporating fsQCA solutions into a regression model via Tobit regression analysis can serve as a post hoc examination to offer extra and complementary perspectives on the phenomenon under investigation [38]. Consequently, this research carried out Tobit regression analysis, using online teaching effectiveness as the response variable and all structural statements pertaining to online teaching effectiveness as the predictor variables. Following the principles of Boolean algebra [38], the configuration statement values need to be transformed from their initial values.

## 5. Results

### 5.1. FsQCA results

The factors of the configuration analysis were shown in Tables 1 and 2. The configuration for online teaching effectiveness consists of three paths: the complex solution, the intermediate solution, and the parsimonious solution. The core factors in the complex and intermediate solutions are critical conditions for achieving effectiveness, while the factors present in the complex solutions but absent in the parsimonious solutions are peripheral conditions. These peripheral conditions are also important for online teaching effectiveness. The three sub-configurations within the complex solutions are based on the same set of core factors (AFT*TPACK) and contribute to a high level of online teaching effectiveness.

If the two core elements based on AFT and TPACK are referred to as the main configuration, then it can be seen from Tables 4 and 5 that all three paths use AFT and TPACK as the core essentials, differing only in terms of the edge conditions, which are CDI, TTS and TSI for the first path, whereas SAL does not have a significantly different impact on the effectiveness of online teaching and learning. The second path had TSI as an peripheral condition and TTS, CDI, and SAL as absent condition.The third path had CDI as an peripheral condition and TSI, TTS, and SAL as absent condition.

**Table 4:** Results of the configuration analysis.

Configurations	Solution
Configurations 1	TPACK*TSI*TTS*CDI*AFT
Configurations 2	TPACK* ~ SAL*TSI* ~ TTS* ~ CDI*AFT
Configurations 3	TPACK* ~ SAL* ~ TSI* ~ TTS*CDI*AFT

Notes: * Means Boolean logic “and”; ~ means Boolean logic “not”.

**Table 5:** Configurations for online teaching effectiveness.

Factor	Online Teaching Effectiveness		
	1	2	3
Acceptability for teachers(AFT)	●	●	●
Course design and implementation(CDI)	●	ⓧ	●
Teaching technological support(TTS)	●	ⓧ	ⓧ
Teacher-student interaction(TSI)	●	●	ⓧ
Students’ autonomous learning(SAL)		ⓧ	ⓧ
Teacher’s TPACK(TPACK)	●	●	●
Raw coverage	0.495504	0.28721	0.303583
Unique coverage	0.20467	0.0143605	0.0418738
Consistency	0.927405	0.938596	0.903715
Solution coverage	0.551738		
Solution consistency	0.900153		

Note: ● indicates the presence of a condition and ⓧ indicates its absence. Major circles denote central conditions, while minor circles denote peripheral conditions. Empty spaces denote neutrality towards the condition.

The solution coverage refers to the extent to which the result of a complete solution term interpretation includes its members. Meanwhile, solution consistency measures how well the members of the solution terms correspond to the members of the result. Based on these two values, the solution demonstrates strong solution coverage and solution consistency.

### 5.2. Complementary analysis results

#### 5.2.1. Sensitivity analysis result.

The results of the sensitivity analysis showed that the resulting solutions and paths were essentially the same as the previous solution terms and paths, despite adjusting the anchors to the upper, middle, and lower quartiles.

#### 5.2.2. Predictive validity result.

**Fig 2:**
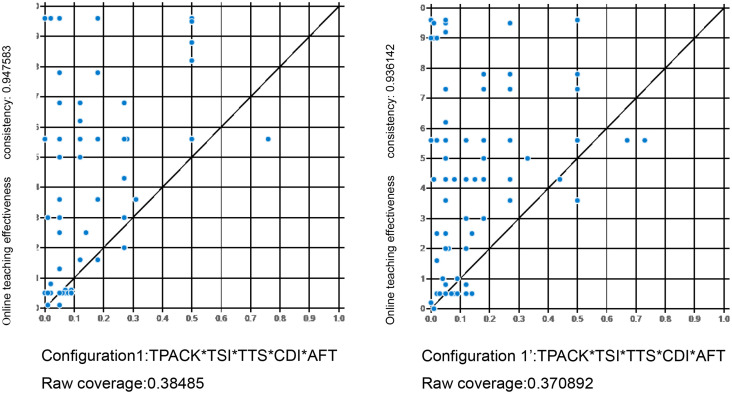
XY scatter-plots of online teaching effectiveness in Configuration1a and 1a’. XY scatter plots of online teaching effectiveness in Configuration 1a and Configuration 1a’Scatter plots depicting the distribution of online teaching effectiveness values for Configuration 1a (left panel, analyzed using sub-sample 1) and Configuration 1a’ (right panel, analyzed using sub-sample 2). The x-axis and y-axis represent the continuous value range of online teaching effectiveness from 0.0 to 1.0, reflecting the variability and distribution characteristics of the outcome variable across the two sub-samples.

In this study, the configuration in sub-sample 1 was tested using the data of sub-sample 2. The consistency and coverage of the results of all model tests were similar (Fig 2 shows the model tests with configurations 1). Therefore, the proposed configuration had a high predictability on different data sets.

#### 5.2.3. Post hoc analysis result.

As can be seen in Table 6, the p *P*-values in all three configurations were <0.05, which indicated that Configuration 1 (*β* = 1.420685, *P* = 0.0000), Configuration 2 (*β* = 0.957893, *P* = 0.0149) and Configuration 3 (*β* = 1.114056, *P* = 0.0028) had a significant effect on online teaching effectiveness. These results correspond roughly to the results obtained by fsQCA.

**Table 6:** Results of the Tobit regression analysis for online teaching effectiveness.

Independent variable	Coefficient	Standard error	z-Statistic	Probability
Configuration 1	1.420685	0.162293	8.753828	0.0000
Configuration 2	0.957893	0.393435	2.434691	0.0149
Configuration 3	1.114056	0.372835	2.988067	0.0028

Notes: *P < 0.1; ** *P* < 0.05; *** *P* < 0.01.

## 6. Discussions

This study utilized fsQCA analysis to investigate the configuration styles influencing elementary school teachers’ online teaching effectiveness (OTE), identifying three distinct configurations. These configurations are not random combinations of conditions but represent context-adaptive mechanisms for achieving high OTE, with teachers’ acceptability of online teaching (AFT) and Technological Pedagogical Content Knowledge (TPACK) emerging as core determinants. The fundamental rationale for this finding lies in the developmental characteristics of elementary students—their limited self-regulation ability means teachers’ internal motivation and professional competence are far more critical to OTE than external resources. This study further identifies core and peripheral conditions within these configurational pathways via fsQCA, clarifying universally essential factors and context-dependent supplementary conditions for OTE, and ultimately providing targeted strategies for elementary schools to optimize online teaching quality in both emergency and routine scenarios, with specific application strategies for each condition elaborated below.

In terms of teachers’ acceptance of online teaching, past research shows most teachers accept online teaching as a temporary substitute for traditional instruction yet perceive it as less effective than face-to-face teaching [4,5]. Nevertheless, AFT is a foundational condition across all three high-OTE configurations, as psychological acceptance is a prerequisite for teachers to invest sufficient effort in online lesson preparation and delivery. The inherent reason AFT is indispensable is that online teaching demands sustained effort, flexible adaptation and proactive engagement in dynamic teaching environments—all of which rely on stable psychological acceptance of virtual instruction. Beyond being a psychological prerequisite, AFT acts as a dynamic driver of adaptive behavior under resource constraints: teachers with high AFT proactively seek alternative solutions when formal support is lacking, directing their effort to compensate for missing peripheral factors [5]. In contrast, low AFT leads to the abandonment of online teaching adaptations, even for teachers with strong TPACK, as AFT functions as a foundational enabler that unlocks TPACK’s value—without it, advanced technological pedagogical knowledge remains underutilized. This motivational mechanism is core to OTE: AFT provides the sustained initiative needed to activate and apply professional knowledge in the complex, uncertain online teaching context. For application strategies, AFT is a foundational prerequisite in both scenarios. In routine settings, schools cultivate AFT through regular experience sharing and professional training to shape a positive attitude toward online teaching as a complement to traditional instruction. In emergency scenarios, timely psychological counseling and peer support are prioritized to alleviate adaptation anxiety and consolidate teachers’ psychological acceptance of remote teaching.

In terms of teachers’ TPACK, TPACK is a core condition across all three configurations, further verifying that the synergistic effects of its three components are crucial for effective online teaching [20,21,23]. Its universal presence reflects the knowledge-intensive and situation-dependent nature of elementary online teaching, which requires dynamic integration of technology, pedagogy and content. TPACK’s role is context-adaptive rather than uniform: in Configuration 1 (TPACK*TSI*TTS*CDI*AFT) with complete peripheral resources, it manifests as integrative competence, enabling teachers to leverage high-quality technological platforms and align them with subject-specific pedagogies [23]. In Configurations 2 and 3 with missing peripheral factors, it shifts to adaptive competence, allowing teachers to modify pedagogical approaches to fit limited tools and compensate for resource gaps. This contextual variability expands TPACK theory, as prior variable-centered studies overlook its adaptive role and focus only on its general importance [21]. The dual role of TPACK—integrative and adaptive—explains the multiple pathways to high OTE, as teachers’ professional competence reshapes its function based on environmental conditions rather than following a fixed pattern. For application strategies, TPACK training is differentiated by scenario. Routine scenario training focuses on systematic integrative competence development, including advanced technology-subject pedagogy integration. Emergency scenario training features targeted, short modules for adaptive competence, such as simple tool application and rapid offline-to-online teaching transition.

The irrelevance or absence of students’ autonomous learning (SAL) across configurations highlights that AFT and TPACK collectively offset the limitations of elementary students’ underdeveloped self-regulation (ages 6–12), a result rooted in young learners’ cognitive and behavioral characteristics of heavy reliance on teacher guidance. SAL is an irrelevant factor in Configuration 1 and a missing factor in Configurations 2 and 3: in Configuration 1, teachers with high AFT and TPACK design scaffolded autonomy activities, where SAL is supported by teacher-led structures and thus its direct impact on OTE is reduced. In Configurations 2 and 3, AFT and TPACK further compensate for absent SAL—high AFT drives extra time investment in asynchronous feedback, while TPACK enables the design of visual, age-appropriate learning materials that minimize reliance on student autonomy. This contradicts Cao et al.’s framing of SAL as a standalone factor [12]; instead, SAL’s role is mediated by AFT and TPACK, which structure the learning environment to fit students’ developmental needs and render SAL non-critical. This substitutive effect reflects a core logic of elementary online teaching: teachers’ active guidance compensates for the natural deficiency of young learners’ autonomous learning ability. For application strategies, routine settings leverage layered scaffolded activities to cultivate SAL as a supplementary condition. Emergency settings rely on AFT and TPACK to fully compensate for absent SAL via increased targeted feedback and structured, visual learning content.

In terms of teacher-student interaction (TSI), previous research confirms TSI’s positive role in student learning outcomes, with synchronous communication being particularly effective for online learning [24–27]. However, TSI is a present condition in Configurations 1 and 2 and a missing condition in Configuration 3, indicating it is less essential than AFT and TPACK. TSI is not universally necessary because its functions can be replaced or transformed by AFT and TPACK-driven instructional behaviors, which shape the quality, not just quantity of interaction and resolve inconsistencies in prior research that emphasizes TSI’s universal value [26]. In Configuration 1 (TSI and TTS present), TSI takes the form of dialogic interaction via synchronous tools for in-depth discussions; in Configuration 2 (TSI present, TTS absent), it shifts to targeted asynchronous interaction using text-based feedback for individual student gaps; in Configuration 3 (TSI and TTS absent), AFT and TPACK enable embedded interaction through pre-recorded lessons with built-in checkpoints, with teachers adjusting instruction based on student responses. This reveals a key causal mechanism: TSI’s contribution to OTE depends on its quality and form, not mere presence, and its necessity is determined by the extent to which AFT and TPACK can replace its functions. For application strategies, routine settings prioritize high-quality dialogic interaction via synchronous tools. Emergency settings with basic interaction channels use targeted asynchronous interaction; extreme emergency settings with no TSI rely on embedded interaction in pre-recorded lessons to replace TSI functions.

In terms of course design and implementation (CDI), prior studies argue online courses require highly organized design, implementation and evaluation [24,30]. Yet CDI is present in Configurations 1 and 3 and missing in Configuration 2, identifying it as a peripheral condition whose functions can be compensated by teachers’ adaptive adjustment and real-time optimization based on student needs. In Configuration 2 (TPACK* ~ SAL*TSI* ~ TTS* ~ CDI*AFT), AFT and TPACK compensate for simplified CDI under resource constraints: high AFT motivates teachers to refine teaching modules based on student feedback (e.g., adjusting pace for low engagement), while low AFT leads to the use of generic, unmodified modules and lower OTE. This highlights that CDI’s core value lies in alignment with student needs, not complexity—an alignment that AFT and TPACK enable even with simplified design. The underlying logic is that high-quality online teaching is determined by responsiveness to students, not the completeness of pre-set course design. For application strategies, routine settings feature systematic, high-quality CDI as a supplementary condition for OTE. Emergency settings simplify CDI on the premise of student need alignment, with real-time optimization based on feedback to compensate for simplified design.

Finally, in terms of technological support for teaching and learning (TTS), some researchers frame technological factors as necessary for smooth online teaching [6,7,13,15], while others argue online pedagogical training should center on teaching and assessment methods rather than just technology [26]. This study validates the latter view: TTS is only present in Configuration 1 and missing in Configurations 2 and 3, confirming it is a supportive but not sufficient or independent factor for OTE, and must serve content and pedagogy to be effective. TTS is non-essential because teachers with strong AFT and TPACK achieve self-supported tech use, reducing dependence on external support. In Configuration 1, high AFT and TPACK drive proactive TTS utilization, while low AFT leads to reactive use and fragmented instruction; in Configurations 2 and 3, AFT and TPACK enable self-supported tech use aligned with subject content, challenging Noesgaard & Ørngreen’s [15] framing of TTS as essential. This yields a critical conclusion: technology support is only effective when activated by teachers’ internal motivation and professional capability, and can be replaced by strong AFT and TPACK under resource constraints. For application strategies, routine settings build a complete professional TTS system and guide proactive use to synergize with other conditions. Emergency settings activate self-supported tech use via targeted simple tool training, enabling teachers to explore alternative technological solutions to replace formal TTS.

## 7. Theoretical and practical implications

### 7.1. Theoretical implications

The theoretical implications of this study lie in its advancement of configurational thinking in educational technology research, addressing the limitations of prior variable-centered approaches that overemphasized linear causality. By identifying teachers’ acceptability for online teaching (AFT) and TPACK as universal core conditions and unpacking their synergistic interactions with context-dependent peripheral factors (e.g., course design, technical support), this research refines the Community of Inquiry (CoI) framework and similar theoretical models by introducing a hierarchical structure of “universal enablers” and “contextual adjusters.” Furthermore, the study resolves inconsistencies in prior literature—such as the debated necessity of student autonomous learning and technological support—by demonstrating that these factors function conditionally rather than independently, providing a new causal explanation paradigm for understanding online teaching effectiveness (OTE) in elementary education. This not only enriches the theoretical landscape of online education research but also validates fsQCA as a powerful tool for unpacking complex educational phenomena that resist linear interpretation.

### 7.2. Practical implications

Building on the identified configurational pathways, the practical implications of this study offer actionable, context-specific guidance for optimizing elementary online teaching. Given that AFT and TPACK emerged as non-negotiable core conditions, the findings highlight the urgency for educational institutions to shift from isolated technical training to integrated TPACK development programs—particularly tailored for lower elementary teachers working with developmentally limited learners—and to mitigate psychological barriers to online teaching via peer mentoring and context-relevant success case sharing. Additionally, the three distinct configurations provide a “roadmap” for resource allocation: urban schools with robust technical support can prioritize the full-factor combination (TPACK*TSI*TTS*CDI*AFT), while rural or resource-constrained schools can focus on the more parsimonious pathway centered on AFT, TPACK, and high-quality course design. This targeted approach avoids the “one-size-fits-all” inefficiency of traditional interventions, enabling schools to optimize OTE based on their unique student demographics, subject mix, and infrastructure conditions.

## 8. Conclusion

This study employed fuzzy-set Qualitative Comparative Analysis (fsQCA) to explore the configurational pathways influencing elementary school teachers’ online teaching effectiveness (OTE) during the COVID-19 pandemic, focusing on three dimensions: human factors (teacher, student, and teacher-student interaction), curricular factors, and technological factors. The key findings reveal that high OTE is not contingent on the presence of all factors, but rather on synergistic configurations anchored in two universal core conditions: teachers’ acceptance of online teaching (AFT) and Technology Pedagogical Content Knowledge (TPACK). All other factors—including student autonomous learning, teacher-student interaction, course design quality, and technological support—function as context-dependent peripheral conditions, exerting conditional rather than indispensable effects. These results enrich the understanding of OTE’s multi-factor dynamics, moving beyond linear causality to highlight configuration-based synergies.

To enhance elementary online teaching effectiveness, targeted recommendations for key stakeholders are as follows, aligned with the study’s findings:(1) For educators: Prioritize strengthening AFT and TPACK to adapt to diverse resource contexts. (2) For policymakers: Develop regionally tailored policies—investing in TPACK training for rural areas with limited TTS and in TTS infrastructure for urban areas—while centering AFT in teacher support initiatives. (3) For schools: Establish peer mentoring systems where teachers skilled in high-OTE configurations share strategies, and curate context-adaptable online teaching resources.

Despite its contributions, the study has notable limitations. First, the sample, while demographically representative of China’s elementary teaching workforce, is geographically restricted to several provinces, potentially limiting generalizability to regions with distinct infrastructure or cultural contexts (e.g., Western China). Second, the inclusion of diverse subject areas may obscure discipline-specific configurational patterns (e.g., pathways for mathematics vs. language instruction). Third, cross-sectional data prevents tracking how OTE configurations evolve with long-term changes in student development or technological advancement.

Future research should address these gaps by expanding regional coverage to incorporate underrepresented areas, conducting subject-specific fsQCA analyses to identify specialized pathways, and adopting longitudinal designs to capture temporal dynamics of causal configurations. Additionally, integrating mixed methods (e.g., follow-up interviews with teachers in high-OTE configurations) could further unpack the mechanisms underlying observed synergies, providing deeper insights for theory refinement and practice optimization. Collectively, this study underscores the value of configurational thinking in OTE research and offers a foundation for targeted interventions to enhance elementary online teaching.

## Supporting information

S1 Fig Conceptual model of online teaching effectiveness. (DOCX)

S2 Fig XY scatter-plots of online teaching effectiveness in configuration 1a and 1a’. (DOCX)
