# Supplementary material for: Optimizing online teaching effectiveness in elementary education: Exploring multifaceted pathways based fsQCA analysis
Source: PLoS One. 2026 Mar 23;21(3):e0345463. doi: 10.1371/journal.pone.0345463 (PMC13008059; doi:10.1371/journal.pone.0345463)
Supplement: S1 Fig — (DOCX) [file pone.0345463.s001.docx]

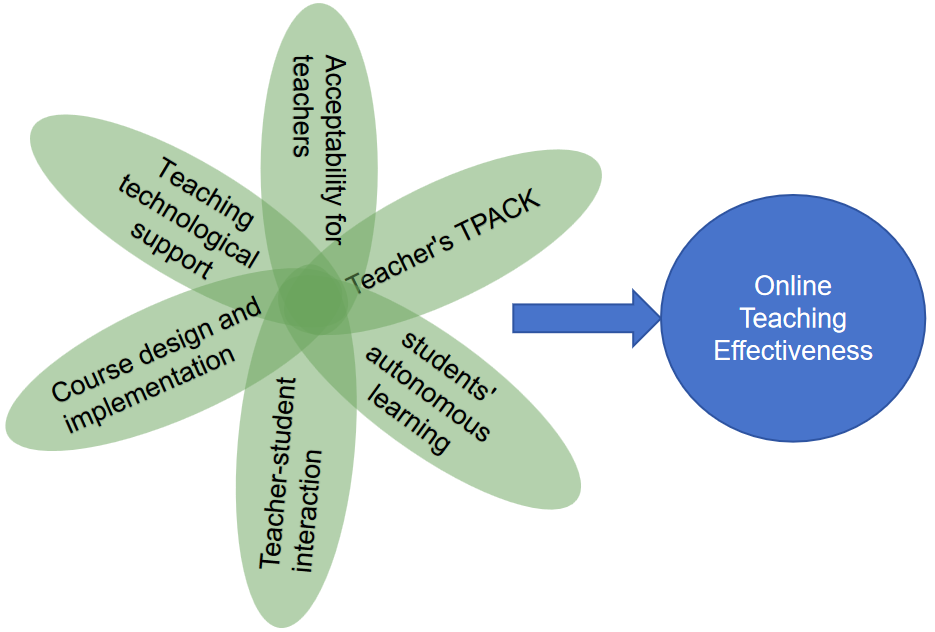


**Fig. 1 Conceptual model of Online Teaching Effectiveness.** This conceptual model illustrates the key antecedent factors and their direct associations with online teaching effectiveness, including teachers’ acceptability for teachers, teaching technological support, online course design and implementation, students’ autonomous learning and teachers’ technological pedagogical and content knowledge (TPACK). All identified factors are posited as core drivers of the effectiveness of online teaching practices.
