# Supplementary material for: Optimizing online teaching effectiveness in elementary education: Exploring multifaceted pathways based fsQCA analysis
Source: PLoS One. 2026 Mar 23;21(3):e0345463. doi: 10.1371/journal.pone.0345463 (PMC13008059; doi:10.1371/journal.pone.0345463)
Supplement: S2 Fig — (DOCX) [file pone.0345463.s002.docx]

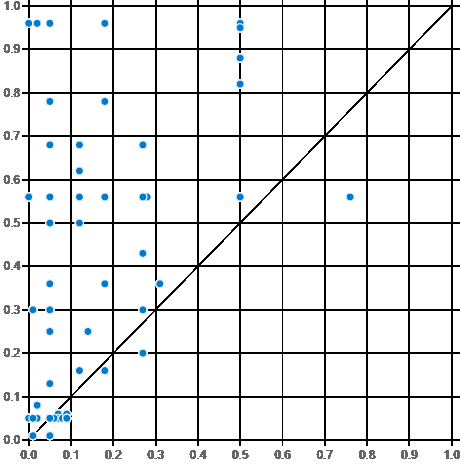

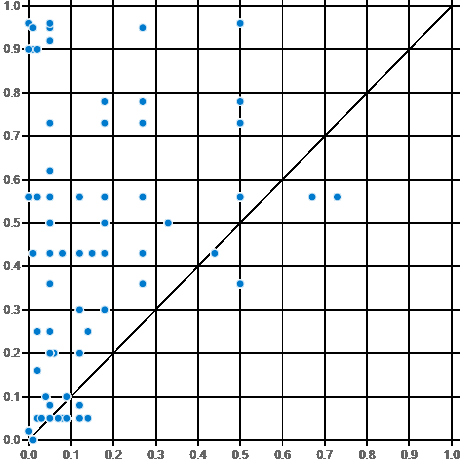


Online teaching effectiveness consistency: 0.936142

Online teaching effectiveness consistency: 0.947583

Configuration 1’:TPACK*TSI*TTS*CDI*AFT

Raw coverage:0.370892

Configuration1:TPACK*TSI*TTS*CDI*AFT Raw coverage:0.38485

**Fig 2: XY scatter-plots of online teaching effectiveness in Configuration 1a and 1a’.** XY scatter plots of online teaching effectiveness in Configuration 1a and Configuration 1a’Scatter plots depicting the distribution of online teaching effectiveness values for Configuration 1a (left panel, analyzed using sub-sample 1) and Configuration 1a’ (right panel, analyzed using sub-sample 2). The x-axis and y-axis represent the continuous value range of online teaching effectiveness from 0.0 to 1.0, reflecting the variability and distribution characteristics of the outcome variable across the two sub-samples.
